# Changing Domesticity of *Aedes aegypti* in Northern Peninsular Malaysia: Reproductive Consequences and Potential Epidemiological Implications

**DOI:** 10.1371/journal.pone.0030919

**Published:** 2012-02-17

**Authors:** Rahman G. M. Saifur, Hamady Dieng, Ahmad Abu Hassan, Md Rawi Che Salmah, Tomomitsu Satho, Fumio Miake, Ahmad Hamdan

**Affiliations:** 1 School of Biological Sciences, Universiti Sains Malaysia, Penang, Malaysia; 2 Faculty of Pharmaceutical Sciences, Fukuoka University, Fukuoka, Japan; Louisiana State University, United States of America

## Abstract

**Background:**

The domestic dengue vector *Aedes aegypti* mosquitoes breed in indoor containers. However, in northern peninsular Malaysia, they show equal preference for breeding in both indoor and outdoor habitats. To evaluate the epidemiological implications of this peridomestic adaptation, we examined whether *Ae. aegypti* exhibits decreased survival, gonotrophic activity, and fecundity due to lack of host availability and the changing breeding behavior.

**Methodology/Principal Findings:**

This yearlong field surveillance identified *Ae. aegypti* breeding in outdoor containers on an enormous scale. Through a sequence of experiments incorporating outdoors and indoors adapting as well as adapted populations, we observed that indoors provided better environment for the survival of *Ae. aegypti* and the observed death patterns could be explained on the basis of a difference in body size. The duration of gonotrophic period was much shorter in large-bodied females. Fecundity tended to be greater in indoor acclimated females. We also found increased tendency to multiple feeding in outdoors adapted females, which were smaller in size compared to their outdoors breeding counterparts.

**Conclusion/Significance:**

The data presented here suggest that acclimatization of *Ae. aegypti* to the outdoor environment may not decrease its lifespan or gonotrophic activity but rather increase breeding opportunities (increased number of discarded containers outdoors), the rate of larval development, but small body sizes at emergence. Size is likely to be correlated with disease transmission. In general, small size in *Aedes* females will favor increased blood-feeding frequency resulting in higher population sizes and disease occurrence.

## Introduction

*Aedes aegypti* is a common domestic vector mosquito, which lives in close association with and shows a preference for feeding on humans, even when other hosts are available [1], [2], [3]. It is considered one of the world's most important mosquito vector species because of its high degree of susceptibility to virus infection [4] and is an efficient epidemic vector of several human diseases, including dengue fever, Chikungunya, and yellow fever [5], [6]. *Ae. aegypti* is a complex species with a combination of sylvan and domestic forms [7], [8]. The latter form originated in North Africa from the South African sylvan form during the expansion of the Sahara Desert [9]. This domestic form was transported to the rest of the world through trade and shipping during the 15th–19th centuries. This form has maintained its domesticity up to 83% in East Africa [10]. Moreover, this is a common domestic mosquito in tropical and subtropical countries [11]. However, in a yearlong survey in the Northern peninsula of Malaysia performed in 2009, more than half of the immature *Ae. aegypti* were collected from outdoor containers. Many of those containers were away from human dwellings, *e.g.*, near roadside food stalls. The acquisition of this outdoor breeding or peridomestic adaptation together with indoor breeding behavior can potentially increase the biting activity of this vector species both indoors and outdoors, which may have important implications for disease transmission. Despite its epidemiological importance, there have been no previous studies regarding this issue in relation to the dengue vector *Ae. aegypti*.

The number of gonotrophic cycles (GCs) of a vector is an indicator of its survivorship, the biting frequency as it bites at least once in a single GC [12], and fecundity [13]. These are also the primary components of the epidemiology of dengue. Fecundity is dependent on reproductive success, which is closely related to body size. Several studies have established a relationship between mosquito size and fecundity in that large females have higher fecundity rates than smaller individuals [14], [15]. The later individuals have been reported to be frequent biters [2], and the biting rate of a vector population is a major indicator of parasite and pathogen transmission [16]. The mosquito's habit of taking more than one blood meal per GC [3], [17], [18] can markedly increase the entomological inoculation rate (EIR) [19]. However, mosquitoes that survive for long periods and complete more GCs can become more efficient vectors. Any changes in this cycle may alter factors that determine the incidence of disease. Under- or overestimation of the numbers of GCs can lead to incorrect conclusions regarding the population dynamics and vectorial capacity. These biological and physiological parameters of a vector, the key regulator of disease epidemics, are influenced by the environmental conditions and can differ between geographical areas [20], [21].

Female *Ae. aegypti* shows a preference for laying their eggs in domestic containers, but may also use rainwater-accumulating containers present in the immediate peridomestic environments [22], [23]. Evidence has been produced which demonstrates that this mosquito species exhibits variable levels of domesticity. A striking example of this is the work from Trpis and Hausermann [10] where they clearly found the existence of differential domesticity: three behaviourally distinct populations of *Ae. aegypti*: domestic, peridomestic and feral. In situations similar to those observed by Trpis and Hausermann [10], we have collected immatures of *Ae. aegypti* from containers near house entrance, in backyard, veranda and even from the flushing tank of a toilet close to a dining room in Malaysia.

In this country, the outdoor adaptation of the principal dengue vector, *Ae. aegypti*, has raised questions regarding whether the gonotrophic activity (GA), fecundity, and fitness of this mosquito are similar to those of indoor adapted forms, as they grow in relatively challenging environments, such as small containers with scarcity of food and water and higher temperatures. The indoor mosquitoes grow in a better larval habitat with greater availability of food in large water storage containers and the adults have ready access to human blood meals. Here, we examined these parameters among three groups of *Ae. aegypti*, *i.e.*, wild strain of female mosquitoes originating from outdoor (FWMs) and indoor (*d0*FWMs) containers from Gelugor, Jelutong, and Air Itam, Malaysia, and the domestic 5^th^ generation daughters (*d5*FWMs) from *d0*FWMs. The latest group was used to check the adaptability and fitness of *d0*FWMs. In the laboratory, *d0*FWMs were reared with nutritious food and adults were given sufficient blood feeding from restrained mice to obtain *d5*FWMs. Under similar conditions, GA and fecundity were reported previously to vary widely in *Aedes albopictus* [24]. However, these parameters may not show significant variation in *Ae. aegypti* as this vector has already become adapted to the indoor environment.

## Materials and Methods

### Occurrence of *Ae. aegypti* larvae in outdoor containers

**Figure 1:**
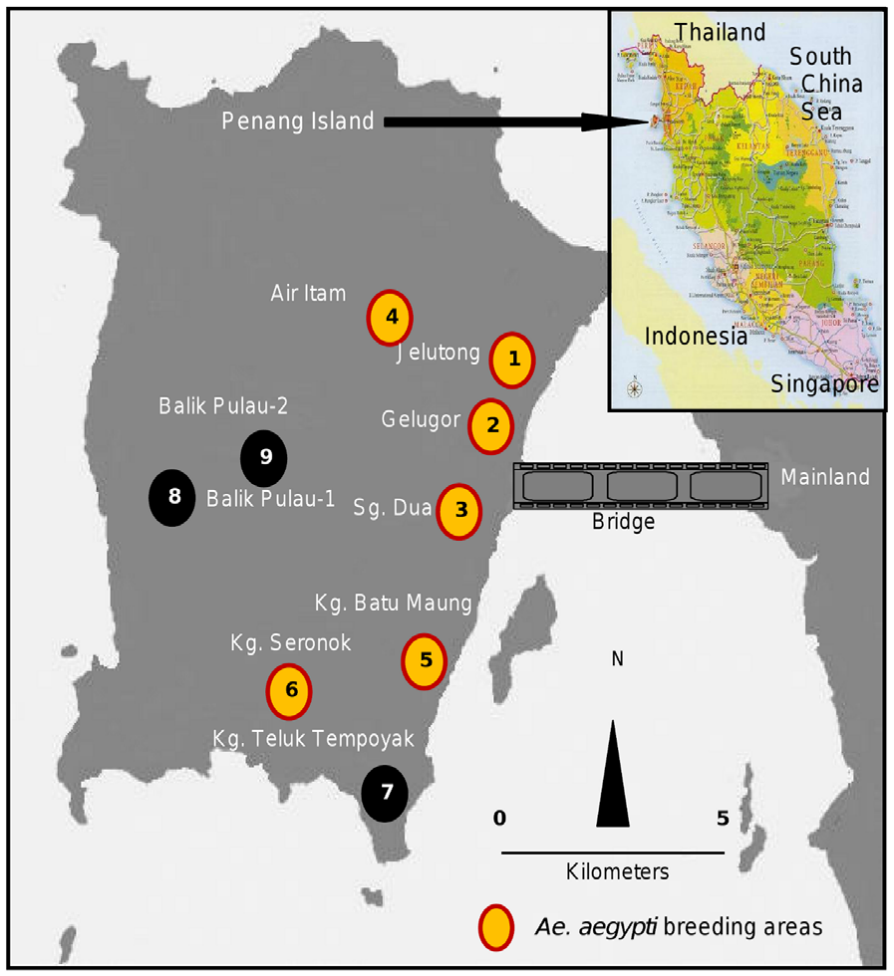
Locations of *Aedes* survey areas on Penang Island, Malaysia.

A yearlong larval survey of *Aedes* mosquitoes was carried out from February 2009 to February 2010 in nine residential areas (townships and villages) on Penang Island, Malaysia, located between latitudes 5°8′N and 5°35′N and longitudes 100°8′E and 100°32′E [25] (Figure 1). The survey areas are low-lying, mostly coastal areas, occupied by human populations and surrounded by hills to the northwest. Immature mosquitoes were collected from the indoor and outdoor containers of the households.

### Ethical issues

This study was carried out in accordance with the principles of the Declaration of Helsinki. The study was approved by the Biological Research Ethics Committee at Universiti Sains Malaysia. The Penang Island City Council approved visiting and the collection of field samples from the homes of city dwellers, and all other necessary permits were obtained for the field studies described in this report.

### Colonization of *Ae. aegypti*

Adult mosquitoes were reared from field-collected pupae. Three separate colonies were maintained from wild pupae collected from miscellaneous outdoor containers (a key outdoor breeding environment), wild indoor pupae collected from indoor drums (a key indoor breeding environment), and laboratory-reared 5^th^ generation mosquitoes in the insectarium at the School of Biological Sciences, Universiti Sains Malaysia, Penang. Larvae were reared routinely on a diet of dried yeast. Adults were fed 10% glucose solution and females were allowed blood meals at two days old on restrained mice. The mosquitoes were colonized under laboratory conditions at a room temperature of 29°C±3°C and relative humidity of 75%±10% (photoperiod 13:10 h, 1-h dusk).

### Bioassays

Bioassays were performed using female mosquitoes derived from wild outdoor mosquitoes (FWMs), females derived from wild indoor mosquitoes (*d0*FWMs), and females derived from *d0*FWMs after five generations (*d5*FWMs) under laboratory conditions.

The oviposition sites for egg deposition in all bioassays consisted of disposable plastic cups (9×11.5 cm) containing 30 mL of tap water and lined with filter papers (#1, Whatman®; Whatman International, Maidstone, UK) as an oviposition substrate. The mosquitoes were given a 10% glucose solution with wads of cotton placed at the top of the cage. The experimental females were given an opportunity to mate prior to every new GC from a rearing cage containing 100 males derived from field-collected pupae.

### Experiments

#### Oviposition responses

The oviposition responses of wild *Ae. aegypti* derived from indoor containers inhabited pupae in relation to blood feeding time were examined in laboratory experiments performed as described previously [24]. The FWMs were fed with 10% glucose solution for two days. Then, a batch of 50 two-day-old females at 09:00 h and another similar batch at 17:00 h were blood-fed on restrained mice. The gravid individuals were placed in individual oviposition cages holding oviposition cups. Egg deposition was checked two days after blood feeding at five time points during the day (09:00, 11:30, 17:00, 20:30, and 23:30). The oviposition cups with eggs were removed and replaced with new cups at each check, and the eggs were counted.

#### Gonotrophic activity and fecundity

This experiment was performed to determine the effects of peridomestication on the number of GCs of outdoor *vs*. indoor *Ae. aegypti* in their lifetimes. Batches of 100 fully blood-fed FWMs, *d0*FWMs, and *d5*FWMs were used in the experiment. The gravid females were placed singly in individual oviposition cups holding a 10% glucose solution. They were repeatedly given access to mating and blood re-feeding in each GC until death. The oviposition substrates were replaced in the oviposition cups in each GC to check whether the generation rank affected the number of egg production times and lifetime fecundity. The wing lengths of all dead test mosquitoes were measured to estimate their body sizes as this parameter was suggested to affect the number of eggs produced.

#### Observation of multiple feeding in *Ae. Aegypti*

Batches of 50 randomly selected FWMs and *d0*FWMs were given the opportunity to take blood meals one each alternate day. They were released individually into the rearing cage holding restrained mice for 20 min, and then both, fed and unfed mosquitoes were returned to the individual oviposition cages for egg laying.

#### Data collection and analysis

Positive Containers (PC) were those found in the field with mosquito larvae. The Container Index (CI) was calculated as the percentage of water-holding containers with immature *Aedes* larvae. Outdoors areas were considered as those outside houses, except forest, in this study. These locations included city and village roads, the areas around shops and gardens, *etc*. Immature stages of field-collected *Aedes* mosquitoes from outdoor and indoor containers were identified according to appropriate taxonomic keys [26]. Females in all bioassays were allowed blood meals for one day and digestion followed by laying eggs for 96 h. Blood feeding was scored based on distention of the abdomen. Number of GCs for each experimental female (FWMs, *d0*FWMs, and *d5*FWMs) and the number of eggs deposited were recorded to score the fecundity as described previously [24]. A GC was considered as the time between ingestion of blood and commencement of oviposition as described previously [27]. At the end of the oviposition period in each GC of FWMs, *d0*FWMs and *d5*FWMs, the oviposited eggs were counted by examining filter papers under a dissecting microscope (Meiji EMZ; Meiji Techno Co. Ltd, Tokyo, Japan). The percentage of surviving females was calculated from the surviving individual and the initial number of females, tested in each GC trial. The oviposition rate was determined as the percentage of females started to lay their eggs in every hour from the initial total number of females. The wing length was measured from the axillary incision to the apical margin excluding the fringe scales under a light microscope (Olympus CX41; Olympus, Tokyo, Japan) [28]. Mosquitoes with wing lengths of 2.9–3.7 mm and 1.7–2.8 mm were considered large and small, respectively. Differences in number of GCs, fecundity, and body size among FWMs, *d0*FWMs, and *d5*FWMs were examined for statistical significance by analysis of variance (ANOVA) performed with the statistical software package SPSS 15.1 (SPSS Inc., Chicago, IL). Tukey's test was applied to separate the means in the fecundity trial. Survival rates were compared based on differences in percentages calculated by descriptive statistics. Oviposition activities of two groups of mosquitoes fed at two different times, number of GCs completed by three different groups of mosquitoes, GC lengths, fecundity, body size, and multiple feeding tendencies were compared by ANOVA. More than one blood meal in a GC was considered multiple feeding. In all analyses, *P*<0.05 was taken to indicate statistical significance.

## Results

### Survey of *Ae. aegypti* outside houses

Six of nine residential areas surveyed were infested with *Ae. aegypti*, and the maximum numbers of positive containers were found outdoors (Table 1). The main outdoor breeding sites of *Ae. aegypti* were miscellaneous containers, including some medium- to large-sized containers, where immature stages were found singly or admixed with *Ae. albopictus*. They were found in almost every month suggesting that oviposition events occur throughout the year. The heterogeneity of larval developmental stages and their persistence supports its successful adaptation to outdoor breeding habitats (Table 2).

**Table 1:** Availabilities of indoor and outdoor *Aedes* breeding containers in the study areas.

Area No	Area Name	Indoor container			Outdoor container		
		No of PCs	CI	Container types*	No of PCs	CI	Container types*
1	Jelutong	16	25	D, CT, B, EPC, EP C	42	28.6	B, EP, AP, EP, PlC, PS, EPCC, S, Db, DC
2	Gelugor	3	13.6	D, W, B, EPC	55	36.2	B, PlC, PS, TP, Tyres, D, PB, Mug, EPC, PS
3	SD and BU	7	15.6	D, CT, B, EPC, EP	54	32.3	PlC, DC, TP, Tr, Ht, B, EPC, Tyres, FV, PB, PS, D
4	Air Itam	8	32	CT, B, AG	101	45.7	EPC, EPCC, PlC, TP,
5&6	Batu Maung	10	17	D, CT, EP, EPC	115	44	DC, CP; EPC, EPCC, PS, D

*SD = Sungai Dua; BU = Batu Uban; PC = Positive container; CI = Container index AG = Ant guards; AP = Artificial ponds; B = Buckets; C = Ceramics; CP = Car parts; CT = Cement tanks; Db = Dustbins; D = Drums; DC = Drum covers; EP = Earthen pots; EPC = Empty paint cans; EPPC = Empty paint can covers; FV = Flower vases; Ht = Helmets; PB = Plastic bowls; PlC = Plastic containers; PS = Plastic sheets; S = Shoes; TP = Tin pots; Tr = Trays; W = Wells.

**Table 2:** Characteristics of *Ae. aegypti* collected from outdoor/peridomestic containers throughout Penang Island in 2009.

Month	Location*	Container types**	First	Second	Third	Fourth	Pupae	Species
12/3/2009	Kg. Batu Uban	PlC, DC, TP, Tr, Ht, Ti	220	320	229	291	109	Mixed
12/4/2009	Gelugor	B, PlC, PS, TP, Ti	224	202	76	199	8	Mixed
18/4/2009	Kg.Seronok	DC	20	33	10	10	2	*Ae. aegypti*
26/4/2009	Jelutong	B, EP, AP	124	212	208	514	40	Mixed
5/5/2009	Kg. Melayu, AI	EPC	10	25	15	0	0	*Ae. aegypti*
13/5/2009	Sg. Dua	B, PlC, EPC, Ti	100	150	70	115	15	*Ae. aegypti*
5/6/2009	Jelutong	B, EP, PlC, PS, EPCC, S, Db	125	248	1283	495	10	Mixed
23/6/2009	Kg.Seronok	CP	10	30	6	5	1	*Ae. aegypti*
15/7/2009	Kg. Batu Maung	EPC	95	140	50	45	38	Mixed
12/8/2009	Gelugor	D, PB, M, B	130	176	78	100	13	*Ae. aegypti*
12/9/2009	Kg. Batu Uban	FV, PB, DC, PS	240	143	90	143	5	*Ae. aegypti*
19/9/2009	Kg. Seronok	EPC	50	80	70	150	20	Mixed
11/10/2009	Gelugor	EPC, PS	50	50	37	60	20	*Ae. aegypti*
15/10/2009	Jelutong	PlC, DC	40	15	33	50	4	*Ae. aegypti*
10/11/2009	Kg. Batu Uban	FV, Ti, B, D	395	770	630	806	100	Mixed
17/11/2009	Kg. Melayu, AI	EPC, EPCC	20	65	63	58	1	*Ae. aegypti*
22/11/2009	Kg. Batu Maung	PS, D, EPC	70	160	320	544	110	Mixed
8/12/2009	Jelutong	B, EPCC	0	12	19	17	3	*Ae. aegypti*

*Sg. = Sungai; Kg. = Kampung; AI = Air Itam; Mixed = immature stages of both *Ae. aegypti* and *Ae. albopictus* were found in the same container.

**AP = Artificial ponds; B = Buckets; Db = Dustbins; D = Drums; DC = Drum covers; EP = Earthen pots; EPC = Empty paint cans; EPPC = Empty paint can covers; FV = Flower vases; Ht = Helmets; M = Mugs; PB = Plastic bowls; PlC = Plastic containers; PS = Plastic sheets; S = Shoes; Ti = Tires; TP = Tin pots; Tr = Trays.

### Oviposition activity of *Ae. aegypti*

**Figure 2:**
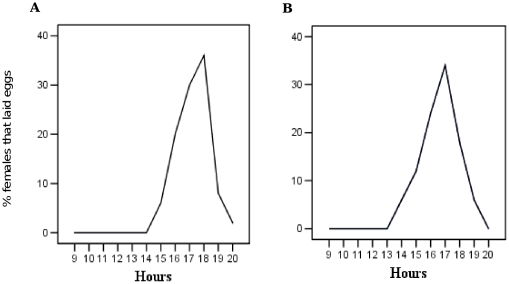
Oviposition responses of FWM *Ae. aegypti* offered blood meals at 09:00 (A) and 17:00 (B).

Figure 2 shows the oviposition responses of *Ae. aegypti* FWMs. Females that took a blood meal in the early morning (09:00, Figure 2A) and in the late afternoon (17:00, Figure 2B) showed only one wide peak of oviposition activity in the afternoon (15:00–19:00). There was no significant difference in egg laying behavior between the two groups of mosquitoes (F = 0.00, df = 1, *P* = 1.000). The maximum number of mosquitoes started to lay eggs between 16:30 and 18:00. In both cases, this activity was low in the evening and absent during the night. Both small- and large-sized mosquitoes started to lay eggs in the afternoon. Small mosquitoes completed egg laying within two to three hours, while larger mosquitoes did not complete egg laying before sunset and started again the following morning, completing the activity before noon.

### Survival and gonotrophic activity period

**Figure 3:**
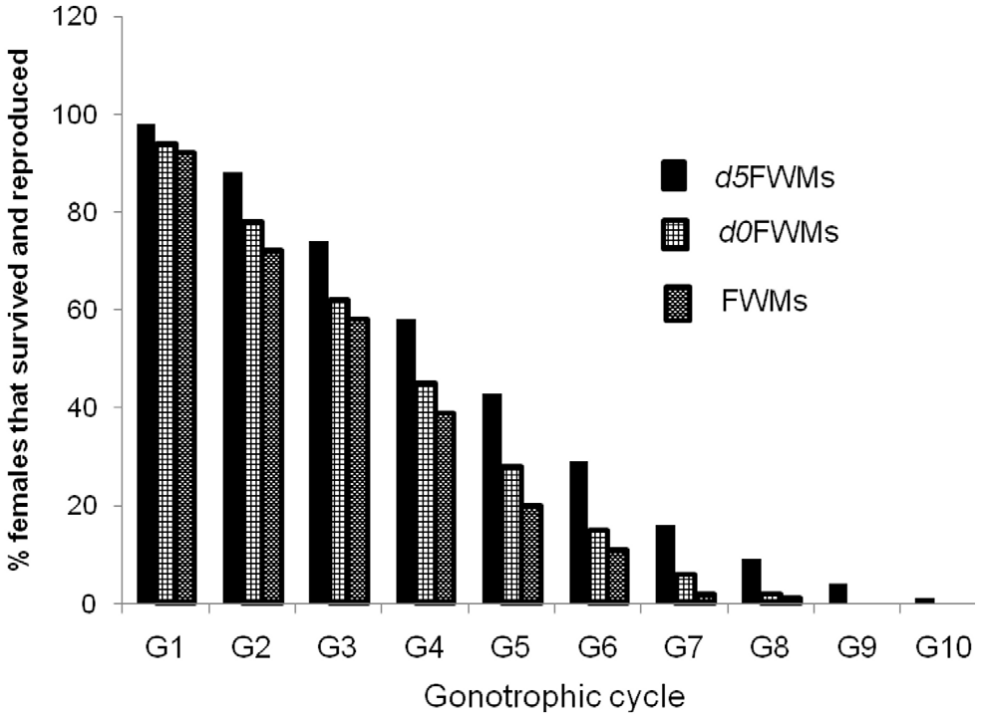
Percentages of surviving FWM, *d0*FWM, and *d5*FWM *Ae. aegypti* and their gonotrophic activity periods.

The survival rates of all three groups of test mosquitoes decreased from first to last GC. This survival pattern varied with female generation. Only one individual from the FWMs survived and achieved an eighth GC, while more than 50% died at the fourth GC. In *d0*FWMs, about 50% of the females died at the fourth GC. Neither of the only two *d0*FWMs that completed an eighth GC showed subsequent survival. More than 50% of females examined from *d5*FWMs survived and attained a fourth GC. About 10% of *d5*FWMs survived and reproduced an eighth time. Among these, one individual reproduced a tenth time. Overall, survival decreased with time, but this was more pronounced among FWMs and *d0*FWMs (Figure 3).

### Gonotrophic activity

**Figure 4:**
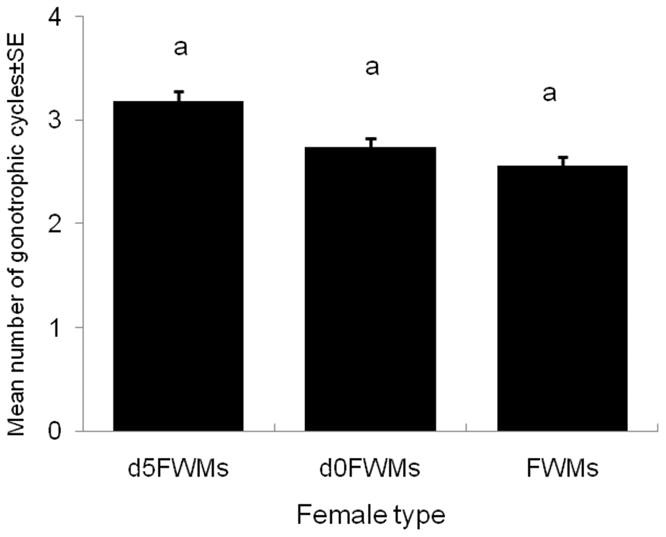
Numbers of gonotrophic cycles (means±SD) of FWM, *d0*FWM, and *d5*FWM *Ae. aegypti*. Bars labeled with the same letter are not significantly different (*P*<0.05).

The number of GCs did not vary significantly among the different generations of *Ae. aegypti* (F = 0.26, df = 2, *P* = 0.78). The numbers of GCs ranged from 1 to 8 in FWMs, *d0*FWMs and from 1 to 10 in *d5*FWMs. The mean number of GCs indicated equivalent fitness of both indoor and outdoor females to reproduce their generation (Figure 4).

### Length of gonotrophic cycle

**Figure 5:**
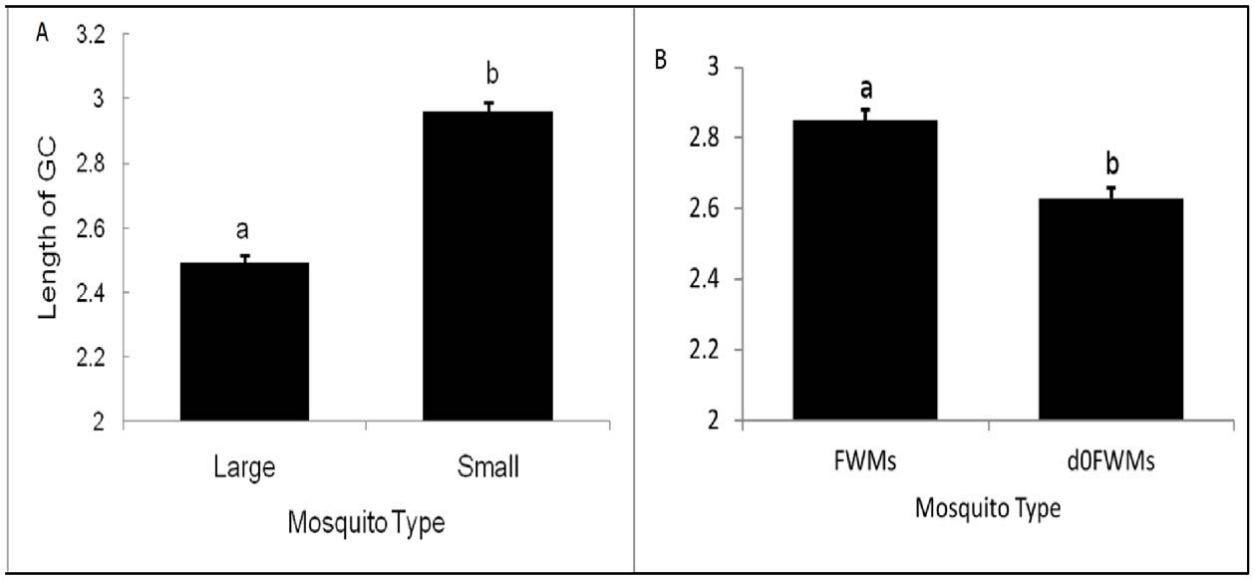
Gonotrophic cycle lengths (means±SD) (A) of large and small *Ae. aegypti*. (**B**) **FWMs and** ***d0*** **FWMs.** Bars labeled with the same letter are not significantly different (*P*<0.05).

The length of the GC varied significantly between large and small mosquitoes (*F*_1,658_ = 155.08, *P*<0.001). The mean GC lengths were 2.49±0.026 and 2.96±0.027 d (range 1.9–4 d) for large and small mosquitoes, respectively. Smaller mosquitoes took a longer time to complete their gonotrophic activity than larger mosquitoes (Figure 5A, Table 3). The GCs of FWMs were significantly longer than those of *d0*FWMs (F = 27.71, df = 1, *P*<0.001) (Figure 5B, Table 3) due to their smaller body size.

**Table 3:** Statistical analysis (ANOVA) of variations in gonotrophic length between A) large and small *Ae. aegypti* mosquitoes, B) Outdoor and indoor *Ae. aegypti* mosquitoes.

	Mosquito Type	df	SS	MS	F	*P*
A	Large	1	36.68	36.68	155.08	0.000
	Small	658	155.65	0.24		
	Total	659	192.33			
B	FWMs	1	6.660	6.66	27.71	0.000
	*d0*FWMs	544	130.74	0.24		
	Total	545	137.40			

### Fecundity

**Figure 6:**
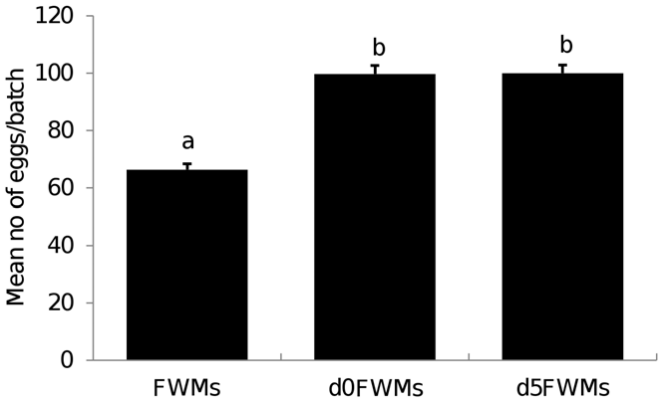
Egg batch size (means±SD) of different types of *Ae. aegypti*. Bars labeled with the same letter are not significantly different (*P*<0.05).

Figure 6 shows the batch size of eggs deposited by different generations of test mosquitoes. Egg batches of similar sizes were produced by *d0*FWMs and *d5*FWMs (*P* = 0.93), while FWMs produced the smallest egg batches (F = 55.51, df = 2, *P*<0.001).

**Figure 7:**
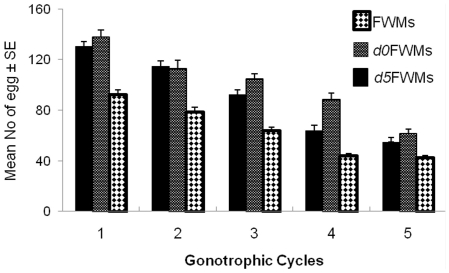
Numbers of eggs (means±SE) laid by different types of *Ae. aegypti*. Bars labeled with the same letter are not significantly different (*P*<0.05).

Figure 7 shows the egg deposition patterns in FWMs, *d0*FWMs, and *d5*FWMs of *Ae. aegypti*. In FWMs, the level of egg production was significantly lower than those of *d0*FWM and *d5*FWM generations except for the fifth GC. However, there were no significant differences in egg production between *d0*FWM and *d5*FWM generations during the first four GCs. The overall significance levels were as follows for the first (F = 7.05, df = 2, *P* = 0.001), second (F = 3.85, df = 2, *P* = 0.02), third (F = 5.35, df = 2, *P* = 0.005), fourth (F = 4.86, df = 2, *P* = 0.009), and fifth GCs (F = 3.87, df = 2, *P* = 0.025).

Egg production tended to decrease as the rank of GC progressed in all generations of females. The first GC in different generations resulted in production of the greatest numbers of eggs; the batch sizes were more than twice as large as those of the fifth GC (Figure 7). The mean numbers of eggs deposited in different GCs were significantly reduced toward the last GC as follows (F = 31.065, df = 4, *P*<0.001), (F = 42.557, df = 4, *P*<0.001) and (F = 62.87, df = 4, *P*<0.001) for FWMs, *d0*FWMs, and *d5*FWMs, respectively, excluding between second and third GC for *d0*FWMs (*P* = 0.27) and the fourth and fifth GCs for FWMs (*P* = 0.768) and *d5*FWMs, (*P* = 0.121) (Table 4). Of the mosquitoes, those that completed up to six GCs laid about 72% of their eggs in the first three cycles.

**Table 4:** Statistical analysis (ANOVA) of variations in fecundity and body size among different generations of *Ae. Aegypti.*

	df	F-ratio	*P*
FWMs	4	42.56	0.000
*d0*FWMs	4	31.07	0.000
*d5*FWMs	4	62.87	0.000
Body size	2	13.11	0.000

### Body size

**Figure 8:**
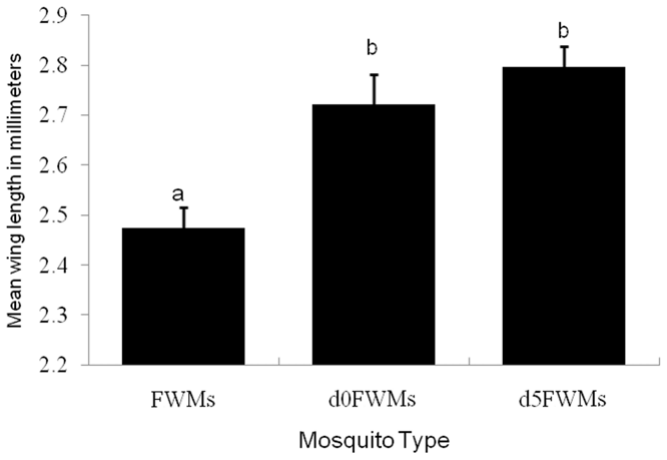
Wing lengths (mean±SD) of different types of *Ae. aegypti*. Bars labeled with the same letter are not significantly different (*P*<0.05).

The mean wing lengths were 2.47±0.04, 2.72±0.06, and 2.80±0.04 mm in FWMs, *d0*FWMs, and *d5*FWMs, respectively. The FWMs were significantly smaller than those in the other two groups (*P*<0.001) but there was no significant difference in mean wing length between *d0*FWMs and *d5*FWMs (Figure 8).

### Multiple feeding tendency

**Figure 9:**
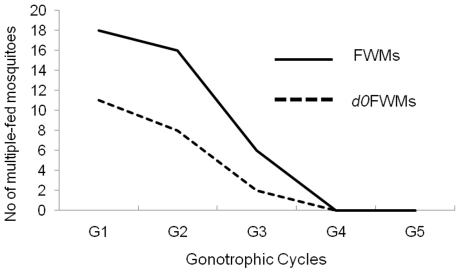
Multiple feeding ratios of *d0*FWM and *d5*FWM *Ae. aegypti* mosquitoes.

The test mosquitoes showed multiple blood-feeding tendencies until the death. This trend was significantly higher in the FWMs than *d0*FWMs (F = 5.87, df = 1, *P* = 0.020). The number of multiple feeding mosquitoes decreased with progression of GCs as the total number of mosquitoes declined (Figure 9).

## Discussion

In this study, we noted more than half of the *Ae. aegypti* breeding containers were outdoors in many areas of Penang Island, Malaysia. The containers were found both immediately surrounding human dwellings and far away from houses. Most were small- to medium-sized rain-filled discarded containers, rather than the more common water containers mainly from indoor habitats [29],[30],[31],[1]. However, the type and nature of the present breeding containers and their numbers were much greater than those in previous studies performed in this area [32], [33], [34]. Moreover, the persistence of heterogeneous immature populations with most developmental stages in small- to medium-sized outdoor containers over a long period suggests that this mosquito has adapted to the outdoor environment. However, there have been no studies regarding the epidemiological and physiological significance of these observations. Here, the gonotrophic performance of wild *Ae. aegypti* collected from indoor and outdoor locations was studied with regard to the crucial interactions between biting activity and fecundity [35], [36].

The egg-laying performance of FWMs and *d0*FWMs was checked under laboratory conditions. Oviposition trials indicated that all generations laid eggs in the laboratory showing a large peak in the afternoon with no correlation with blood feeding time, and the observations were consistent with the natural oviposition behavior of this mosquito species [37], [38], [39], [40]. Gonotrophic activity and fecundity of FWMs and *d0*FWMs were compared with the highly domesticated *d5*FWMs to evaluate the level of domesticity. The latter two groups (*d0*FWMs and *d5*FWMs) showed similar behavior in all experiments, which indicated their similar status in domesticity.

The results of the long-term laboratory study with regard to the number of GCs— a vital indicator of lifespan and vector capacity—indicated that FWMs have maintained their fitness although their fecundity and body size were reduced compared to the other two groups of mosquitoes. Body size and fecundity are dependent on various intrinsic and extrinsic factors that vary between different environments, and natural populations of *Ae. aegypti* are heavily influenced by habitat conditions. The availability of larval food is one of the most important factors dictating immature mosquito body size [41]. Outdoor containers are smaller in size than the common indoor containers, with a limited food supply and larger numbers of immature mosquitoes within a competitive environment [42], [43]. The faster growth due to higher temperatures [44] may not allow enough time to accumulate sufficient energy, or the shift from a simple to a complex wild environment where some extra energy is required to search for shelter and a suitable breeding place may result in physiological changes causing a reduction in body size.

Fecundity is positively correlated with mosquito body size [24]—larger mosquitoes produce more eggs than smaller ones, and those in the field tend to become small, which is also a common observation for field adults of *Ae. aegypti* [45], [46]. However, *Ae. aegypti* also breed in large containers outdoors, such as water storage drums and large buckets, which are commonly seen in this region [47], [48], [49] as well as in the present study areas, where food is available and water is more persistent and these environmental factors may result in production of mosquito generations with large body size. The fecundity of these mosquitoes may be similar to those of *d0*FWMs and *d5*FWMs.

On the other hand, comparatively small- to medium-sized mosquitoes with lower fecundity can also play a role in the transmission of viruses in an ideal environment like the present study areas. Furthermore, the higher outdoor temperature accelerates gonotrophic activity and development of immature mosquitoes. The mosquito GC lasts 2–5 days at an ideal temperature of 25°C–30°C [50], which is extended to ∼10 days at 20°C [51]. The combined larval and pupal development takes ∼6 days at 30°C, which increases to 9 days at 25°C, 11 days at 20°C, 20 days at 15°C, and 23 days at 14°C [52], [53]. As larval development is highly temperature-dependent [54], [55], [56], mosquitoes prefer to lay their eggs in warm water in sun-exposed containers than in shaded containers to support more generations over a given period [57]. Moreover, more rapidly developing immature mosquitoes have less exposure to chemicals, parasites, and predators, as well as reduced risks of desiccation [58]. In addition, higher temperature [59], [60] results in a faster GC [61], thus allowing the mosquitoes to complete more cycles within a short lifespan. Warmer temperatures shorten the extrinsic incubation period (EIP) of viruses, which can increase the rate of dengue transmission [62], [63].

Small mosquitoes feed frequently to compensate for their nutritional lack as well as to produce eggs as females convert about 20% of the ingested blood meal into egg constituents [42]. Smaller females require larger blood meals than larger females for egg production and development [64], which they cannot consume at a single feed as their meal sizes are smaller (1.6–2.5 µL) than those of larger females (2.6–3.5 µL) [65], [4], and therefore they show a higher frequency of biting. These mosquitoes may require more frequent meals to survive in the comparatively complex outdoor environment [66], [39], [67], [40]. In addition, *Ae. aegypti* generally takes more than one blood meal during a GC [1], [18], and the smaller females engage in multiple feeding more often than the larger ones [45]. This tendency may increase among the natural outdoor small mosquitoes, which we found in the present study, where the smaller FWMs took multiple blood meals more often than *d0*FWMs. This multiple feeding behavior increases their contact with human hosts, increasing the opportunities for virus transmission [19], [2], [68], [69], [70].

The current study was carried out basically to systematically document the changing breeding ecology of *Ae. aegypti*, identify the reproductive consequences and forecast the potential epidemiological implications. The study has shown increased outdoor breeding of the mosquito species—the vast majority of immature stage populations were collected from miscellaneous containers located far from homes. Both the larval and pupal stages were present in quite high numbers throughout the survey period. Our results highlight the adaptive capacity of *Ae. aegypti* and essentially evidences the acquirement of an increased outdoor breeding behavior by this principal mosquito vector of dengue viruses. Even though we did not assess the causes of this breeding shift from indoors to outdoors, it could be the consequences of the acquisition of indoor-breeding behavior by the closely related, ecologically similar species, *Ae. Albopictus* [24]. It is possible that the outdoor breeding observed represented a strategy to avoid competition with *Ae. albopictus*, known to have a competitive advantage over a number of other mosquito species including the one studied here [71]. Breeding outdoors may increase population density simply because some socio-economic human behaviors may provide increased breeding opportunities. Due to socio-cultural heritage, Penang people spent most of their daytime outside homes, taking their meals at the many canteens, restaurants, and roadside food stalls [72], [73]. Evidence exists that being outdoors during daytime increases exposure to bites and thus the risk of dengue infection [74]. These societal behaviors trigger also increased number of containers being discarded around public places. *Ae. aegypti* breeds primarily in man-made containers, but also any items that can collect rainwater [75]. With the equatorial climate of Penang, which is characterized by a high rainfall [25], the dengue vector is predicted to have increased breeding opportunities outdoors and some levels of population density maintenance, thereby potentially keeping up transmission risks.
